# Dissecting host stress responses for predictable heterologous gene expression in *E. coli*

**DOI:** 10.1093/nar/gkag256

**Published:** 2026-03-30

**Authors:** Christoffer Rode, Felix Beulig, Mathias Jönsson, Morten H H Nørholm, Leonie J Jahn, Daniel C Zielinski, Bernhard O Palsson, Emre Özdemir, Lei Yang

**Affiliations:** Department of Bioengineering, University of California San Diego, La Jolla, CA 92093, United States; Department of Bioengineering, University of California San Diego, La Jolla, CA 92093, United States

## Abstract

Predictable expression of heterologous genes remains a key challenge in biotechnology, largely due to cellular stresses imposed on the production host. Here, we systematically dissect stress responses in *Escherichia coli* MG1655 expressing diverse proteins under varying promoters and translation efficiencies. Using independent component analysis on new and existing large transcriptomic datasets, we identify distinct responses to transcriptional and translational stresses: excessive heterologous messenger RNA (mRNA) triggers a cold shock response that controls mRNA stability (*cspA–I, deaD*), while protein production activates a heat shock response involving proteolysis and chaperone functions. We further identify a broad adaptation response, consistently co-activated with the heat shock response during protein production, that provides stationary phase regulation (*rspAB*) and osmoregulation (*betABIT*). Targeting these latter functions through strain and media modifications significantly increases eGFP production. Other host stress responses depend on the protein being expressed; e.g. we find production of cysteine-rich proteins to uniquely activate functions regulating iron- and redox homeostasis and oxidative stress responses. This work demonstrates a holistic, systems-level view of cellular stresses to heterologous gene expression by considering transcriptional, translational, and product-specific contributions, paving the way toward predictable and optimized expression strategies.

## Graphical Abstract

**Graphical Abstract:**
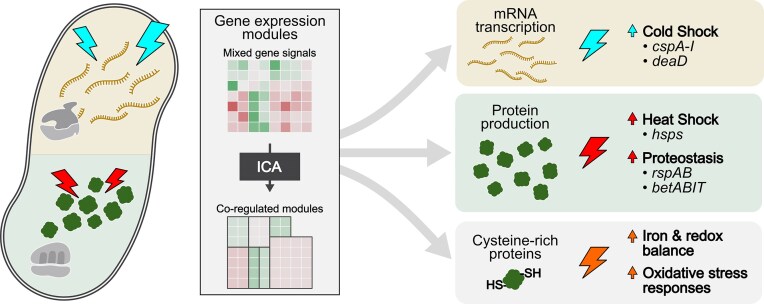

## Introduction

Heterologous gene expression is widely used for producing pharmaceutical proteins [1], industrial enzymes [2], and novel protein products [3]. Here, expression hosts are employed to overproduce a target protein to high levels. Various microbial hosts are available, with *Escherichia coli* being a preferred choice in terms of simplicity, rapid growth, and well-characterized genetics [4]. However, achieving reliable and successful production of proteins remains a challenge. Several factors contribute to failure, including protein product misfolding [5, 6], toxicity [7], size [8], amino acid bias [9, 10], and abiotic factors such as pH, temperature, and osmolarity [11, 12]. Additionally, messenger RNA (mRNA)-related factors also impact expression outcomes, such as codon usage [13], mRNA secondary structures [14], and ribosome binding efficiency [15]. Various strategies can be employed to improve yields and folding of the target protein, such as changing the expression system, optimizing the process conditions, or modifying the product itself [16]. Yet, these strategies largely rely on trial and error due to a limited understanding of expression systems.

A key challenge is the metabolic burden and cellular stress imposed on the host, which can severely impact growth and productivity [17]. Transcription and translation of the target gene consume finite resources, notably nucleotides and charged transfer RNAs (tRNAs), and occupy RNA polymerases and ribosomes [18]. Transcriptional burden is particularly concerning in popular T7 RNA polymerase (T7 RNAP) expression systems, which transcribe high levels of mRNA [19, 20]. While protein misfolding triggers well-known cellular stress responses, including the heat shock response [21], stress responses to transcription of high levels of heterologous mRNA have not been described. Furthermore, how host responses vary with the properties of expressed proteins is unclear. Addressing these knowledge gaps is essential for engineering more predictable expression systems.

Machine learning applied to large -omics datasets offer a powerful approach to describing cellular responses. Independent component analysis (ICA), a signal extraction method, has been successfully developed to deconvolute transcriptomic data into independently modulated gene groups, called iModulons, that correspond to specific cellular functions [22]. In contrast to regulons, iModulons quantify the contribution of genes to regulatory modules by assigning weights to individual genes. Furthermore, iModulons are purely based on transcriptomic data and require no prior knowledge of regulatory networks, and can therefore be used for unbiased discovery of new regulatory modules. iModulon analysis has been used for rapidly revealing the transcriptional regulatory network of diverse organisms [23, 24], identifying novel gene-to-phenotype relations [25], and facilitating the transfer of functions across species [26].

In this study, we investigated how different aspects of heterologous gene expression contribute to cellular stress in *E. coli* expression hosts. To do this, we designed expression strains and obtained transcriptomes while heterologous genes were actively expressed. By leveraging the large PRECISE-1K *E. coli* transcriptome compendium [27] and applying ICA, we interpreted changes in the modularized host transcriptome. This revealed differentially activated iModulons, corresponding to native host responses, that were triggered by various aspects of heterologous gene expression. We referenced existing transcriptome profiles to contextualize these host responses and validated the beneficial roles of novel ones through strain and media engineering. Taken together, this work provides a holistic view of cellular stresses associated with heterologous gene expression and presents new insights toward improving protein production.

## Materials and methods

### Plasmid cloning

Plasmids were constructed via USER assembly [28] and Gibson assembly. Cloning was used to insert heterologous genes into multiple cloning sites, modify RBS sites, integrate an expression site to co-express *rspAB*, and modify CRISPR guide RNA (gRNA) plasmids for genome editing. Monocistronic RBS sequences were selected from the library provided by Mutalik *et al.* [29] and cross-validated with the RBS prediction tool from Salis *et al.* [15]. Unless otherwise specified, transgenes were expressed with a strong bicistronic RBS element, RBS-3 [29]. All expressed transgenes were codon-optimized (Integrated DNA Technologies) and heterologous gene sequences were ordered as gBlocks (Integrated DNA Technologies).

Most plasmids were constructed via USER assembly. Purified fragments were combined in a 10 µl USER reaction containing 1 µl USER Enzyme (New England Biolabs, M5505), 1 µl T4 DNA Ligase Buffer (New England Biolabs, B0202), and insert and backbone fragments at a 3:1 mass ratio. Reactions were thermocycled to excise uracil overhangs, and 4 µl of each reaction was directly transformed into chemically competent *E. coli* TOP10 cells (Invitrogen, C404003) prepared in KCM buffer [30]. Following a 1 h recovery in 250 µl LB medium (37°C, shaking), cells were plated on selective LB agar (ampicillin or kanamycin). Plasmids were isolated from overnight cultures using the QIAprep Spin Miniprep Kit (Qiagen, 27104).

A selection of plasmids was assembled through Gibson cloning. gBlocks were ordered with sequences complementary to vector backbones and assembled using the Gibson Assembly Master Mix (New England Biolabs, E2611). Assemblies were transformed into *E. coli* TOP10 cells and selected on appropriate antibiotics.

### Strain construction

The *E. coli* K-12 MG1655 variant Gly2 (SDT492) was used as the parent strain for developing expression hosts. Gly2 grows optimally on glycerol and carries the following mutations: *yegE* S683Y, *glpK* L65M, *rpoC* L770R [21]. Two knockout derivatives were generated: Gly2 Δ*rhaBADM* (SDT507) and Gly2 Δ*rhaBADM* Δ*rspAB* (SDT1108).

Knockouts were introduced using CRISPR/MAD7-mediated λ-Red recombination. Cells were first transformed with a plasmid expressing the MAD7 endonuclease and arabinose-inducible λ-Red recombination proteins Exo, Beta, and Gamma. To target the genomic deletion site, cells were subsequently co-transformed with a second plasmid encoding a gRNA specific to the locus, a compatible CRISPR RNA, and a repair oligonucleotide for homologous recombination. Transformants were selected on ampicillin and chloramphenicol prior to plasmid curing. SDT507 was counterselected on minimal M9 medium with rhamnose as the sole carbon source to confirm loss of the *rhaBADM* operon. The genome of SDT507 was sequenced and revealed a Δ4500 bp deletion of the *rhaBADM* operon, including a Δ16.4 Kbp spontaneous deletion of a transposable element between genes *yhiM–yhiS*. To construct SDT1108, Cas9 was used in place of MAD7, but following the same procedures.

### Shake flask cultivation

Heterologous gene expression was carried out in 250 ml shake flasks containing 40 ml minimal M9 medium at 37°C with shaking at 225 rpm. Glycerol (0.2% v/v) was used as the sole carbon source, and the media were supplemented with appropriate antibiotics, Wolfe’s vitamin solution (0.1% v/v), and an iron-rich trace metal solution (0.05% v/v). Main cultures were inoculated from pre-cultures (15 ml, 16–17 h) grown from glycerol stocks (OD_600_ = 1) via two 1:100 dilutions to ensure consistency at inoculation. Main cultures were induced at OD_600_ = 0.40 with 1 mM rhamnose, or 1 mM Isopropyl β-d-1-thiogalactopyranoside (IPTG) for pET-vectors. Samples for RNA-seq, OD_600_, and western blot analysis were collected 2 h post-induction.

### Western blotting

Protein production was verified by western blotting (Supplementary Fig. S2). Cell pellets were lysed by heating at 95°C for 15 min in a reducing buffer [90% Invitrogen Laemmli buffer (Bio-Rad) and 10% NuPAGE (Invitrogen)]. Lysates were separated on precast Bio-Rad Protean sodium dodecyl sulfate–polyacrylamide gel electrophoresis gels at 120 V for 5 min, followed by 200 V for 30 min. Proteins were transferred to iBlot membranes using the iBlot 2 Western Blot Transfer System (Thermo Fisher).

Membranes were blocked in skim milk solution (50 g l^−1^ skim milk powder in TBS-T) for 1 h at 4°C, then washed in TBS-T (three rounds, 5 min). Primary anti-His antibody (05–949, Merck), diluted in skim milk solution, was added and incubated overnight at room temperature. Blots were washed as above and incubated for 1 h at room temperature with HRP-conjugated secondary antibody (GENA9310, Cytiva) in skim milk solution. Chemiluminescence was then developed on the blots by adding ECL Western Blotting Detection reagents (Cytiva) and incubating for 3 min in darkness.

### Flow cytometry

eGFP production was quantified from single-cell fluorescence using a NovoCyte Quanteon Flow Cytometer (Agilent). Samples were diluted 1:200 in cold phosphate buffered saline prior to analysis. To filter debris, forward scatter and side scatter thresholds were set to 7500 and 5000, respectively, and 10 000 events were measured per sample. Fluorescence intensity was reported as Median FITC-A. For samples without green cells (>99.5% below threshold), fluorescence intensity was reported as the mean FITC-A across all 10 000 events.

### RNA sequencing

RNA-seq libraries were generated in biological duplicates for all conditions. Total RNA was extracted from cultures according to the RNAprotect Bacteria Reagent Handbook (Qiagen). Cell culture was mixed with RNAprotect Bacteria Reagent (Qiagen) in a 1:2 volume ratio, vortexed, and incubated at room temperature for 5 min. Pelleted cells were resuspended in 400 µl elution buffer, split into two aliquots, and stored at −80°C. Total RNA was extracted and purified using the RNeasy Protect Bacteria Mini Kit (Qiagen) on a QIAcube Connect (Qiagen). Lysozyme was used for the enzymatic lysis of cells, together with Proteinase K for degrading protein, and SUPERase•In RNAse inhibitor (Thermo Fisher) for protecting RNA. DNase treatment was included to remove DNA contamination. RNA was eluted in 30 µl RNase-free water, stored at −80°C, and shipped to Azenta Life Sciences for RNA-seq.

### Gene expression data processing

RNA sequencing data were processed using the pipeline implemented in the iModulonMiner workflow [22]. Raw RNA sequencing data files (FASTQ) were mapped to the genome of *E. coli* K-12 substr. MG1655 and coding sequences on associated expression plasmids. Plasmid gene expression was analyzed separately from transcriptomes. Plasmid gene expression levels were defined as transcript percentage levels relative to the entire transcriptome as follows:


\begin{eqnarray*}
\mathrm{transcript}\ \% = \ \frac{{\mathrm{ FPK}\ \mathrm{ of}\ \mathrm{gene}}}{{\mathrm{FPKs}\ \mathrm{ all}\ \mathrm{genes}}} \times 100.
\end{eqnarray*}

Where FPK is the fragment count normalized by gene length. For transcriptomic analyses, plasmid gene counts were removed, and chromosomal gene counts were normalized to transcripts per million (TPM) and log-transformed (log-TPM).

### Independent component analysis

iModulon activities were calculated via ICA on the combined datasets of PRECISE-1K [27] and our newly generated RNA-seq dataset. Before this, the log-TPM data within each project was centered to project-specific control conditions, and the project “minicoli” was excluded from PRECISE-1K. To run the ICA, iModulonMiner implements the FastICA algorithm using Scikit-learn (v0.20.3), and we used 100 iterations and a convergence tolerance of 1e-7 [22]. To determine the ideal number of components, we used the OptICA method, with a maximum of 370 dimensions and a step size of 20 [31]. We repeated the entire process 100 times to ensure that the final calculated components were robust. The ICA resulted in 156 iModulons. Many iModulons correlated with those in PRECISE-1K, and their descriptions were transferred to our newly calculated iModulons. One novel iModulon was highlighted that we characterized as Proteostasis, which is most closely related to the UC-9 iModulon in PRECISE-1K. The gene membership threshold setting for this iModulon was manually increased to weight = 0.091, to separate low-weight genes that were hovering at its calculated threshold.

### iModulon analysis

Gene expression (log-TPM) values were centered to controls in the dataset; therefore, positive iModulon activity indicate activation, and negative values indicate repression of that iModulon relative to controls. iModulon activities were calculated from gene expression data as the matrix product of gene expression and gene weights:


\begin{eqnarray*}
A = X \times M,
\end{eqnarray*}

where *A* is the iModulon activity matrix, *X* is the log-TPM gene expression matrix, and *M* is the gene weight matrix. For a given iModulon in a sample, the activity is calculated as:


\begin{eqnarray*}
{{A}_{\mathrm{iModulon},\mathrm{sample}}} = \mathop \sum \limits_{\mathrm{gene} = 1}^n {{X}_{\mathrm{gene},\mathrm{sample}}} \times {{M}_{\mathrm{gene},\mathrm{iModulon}}}.
\end{eqnarray*}

The Pymodulon Python package was used to analyze iModulon gene weights and activities [22]. iModulon gene weights were investigated by plotting gene weights across genomic positions and labeling genes exceeding iModulon membership thresholds. Overlapping gene membership between two iModulons was evaluated using scatter plots of gene weights, where genes exceeding the membership threshold in either iModulon were labeled. Coordination between two iModulons across conditions was assessed by pairwise analysis of two iModulon activities, called phase-planes.

### Statistical analysis

All RNA-seq conditions were done in biological duplicates. Hierarchical clustering of transcriptomes was performed using Ward’s method on mean gene expression values within unique conditions (scipy.cluster.hierarchy v1.14.1). Global gene expression analysis was performed to identify differentially expressed genes (DEGs) using the Mann–Whitney U test (scipy.stats v1.14.1). Q-values were derived from *P*-values using the Benjamini-Hochberg procedure to control the false discovery rate (statsmodels v0.14.4). Genes with *q*-values < 0.05 and |log-TPM > 2| were classified as DEGs. Individual gene expression comparisons between groups were performed using Student’s *t*-test (scipy.stats v1.14.1). Linear correlations between protein production/mRNA levels and iModulon activities were assessed using Pearson correlation (scipy.stats v1.14.1). Protein production levels (eGFP fluorescence intensity) at the final timepoint were compared between conditions using Student’s *t*-test (scipy.stats v1.14.1). Ellipses in phase-plane plots represent 2-sigma confidence regions and were calculated from the covariance of each cluster (numpy.linalg.eigh v1.23.5). COG (Clusters of Orthologous Groups) enrichment between RpoH regulon and RpoH iModulon gene sets was evaluated by Fisher’s exact test (scipy.stats v1.14.1). All statistical analyses were performed in Python.

## Results

### Workflow for identifying cellular stress response modules to heterologous gene expression

**Figure 1.**
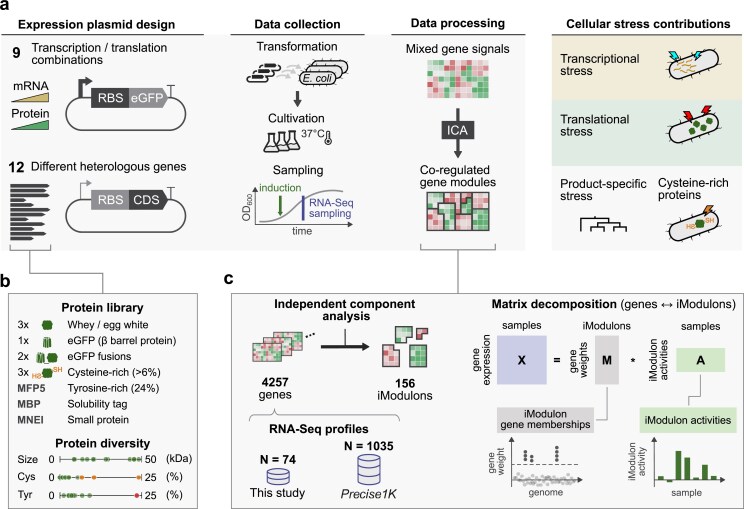
Workflow for identifying cellular stress responses to heterologous gene expression. (**a**) Experimental workflow. Expression plasmids were transformed into *E. coli* and cultivated at 37°C. RNA-seq samples were collected following induction, here illustrated as a marked induction timepoint on a growth curve. iModulon signals were extracted from transcriptomes using ICA, enabling characterization of transcriptional, translational, and product-specific stress contributions, each illustrated as cell icons. (**b**) Design of protein library. The library consisted of 12 different heterologous proteins spanning 0–50 kDa with varying cysteine (0%–25%) and tyrosine (0%–25%) content. Proteins include whey/egg white, eGFP and eGFP fusions, cysteine-rich proteins, tyrosine-rich protein (MFP5), and proteins that fold well (MBP, MNEI). (**c**) ICA methodology. ICA decomposes a gene expression matrix (**X**) into gene weights (**M**) and iModulon activities (**A**), represented as blue, gray, and green rectangles. Gene weights define the membership of genes to each iModulon and relate gene expression to iModulon activities gene expression to iModulon activities across samples (*X* = M*A). RNA-seq dataset from this study (*N* = 74) was combined with the PRECISE-1K dataset (*N* = 1035) to calculate 156 iModulons from 4257 genes.

A systematic workflow was set up to dissect cellular stress responses to heterologous gene expression (Fig. 1a). Expression plasmids were designed and transformed into an *E. coli* MG1655 host, and RNA-seq samples were collected to profile transcriptional responses.

First, to separate the effects of mRNA transcription from translation, we engineered plasmid expression systems that decouple mRNA transcription and protein production. This was achieved by expressing enhanced green fluorescent protein protein (eGFP [32]) under variable ribosome binding site (RBS) strengths in native and T7 RNAP systems.

Second, to investigate protein production stress, we constructed expression plasmids encoding 12 additional proteins that varied in size (8.6–48.0 kDa), folding properties, and amino acid composition (Fig. 1b). This library included globular proteins (whey proteins α-lactalbumin and β-lactoglobulin, egg white protein ovalbumin), fusion proteins (eGFP-ALA and eGFP-BLG), proteins that fold well (*E. coli* maltose-binding protein [33] and monellin derivative MNEI [34]), and proteins with biased amino acid compositions. Three selected proteins are rich in cysteine [thaumatin II (6.6% cysteine), brazzein (10.4%), and metallothionein-1 (24.1%)], while mussel foot protein-5 is rich in tyrosine (23.4%), glycine (19.4%), and lysine (18.3%). For information on protein sequences, amino acid contents, and expression plasmid maps, see “Data availability” section.

To identify cellular responses activated in our heterologous gene expression designs, we applied ICA to new and existing transcriptomic data. ICA is a signal extraction method that decomposes gene expression into independently modulated gene sets (iModulons). iModulon activity corresponds to the weighted expression of its member genes (see “Materials and methods” section for details). We generated a total of 74 transcriptomes in this study and integrated them with 1035 transcriptomes from the PRECISE-1K *E. coli* RNA-seq compendium [22, 27]. We then applied ICA on this combined dataset, decomposing 4257 genes into 156 distinct iModulons (Fig. 1c).

**Figure 2.**
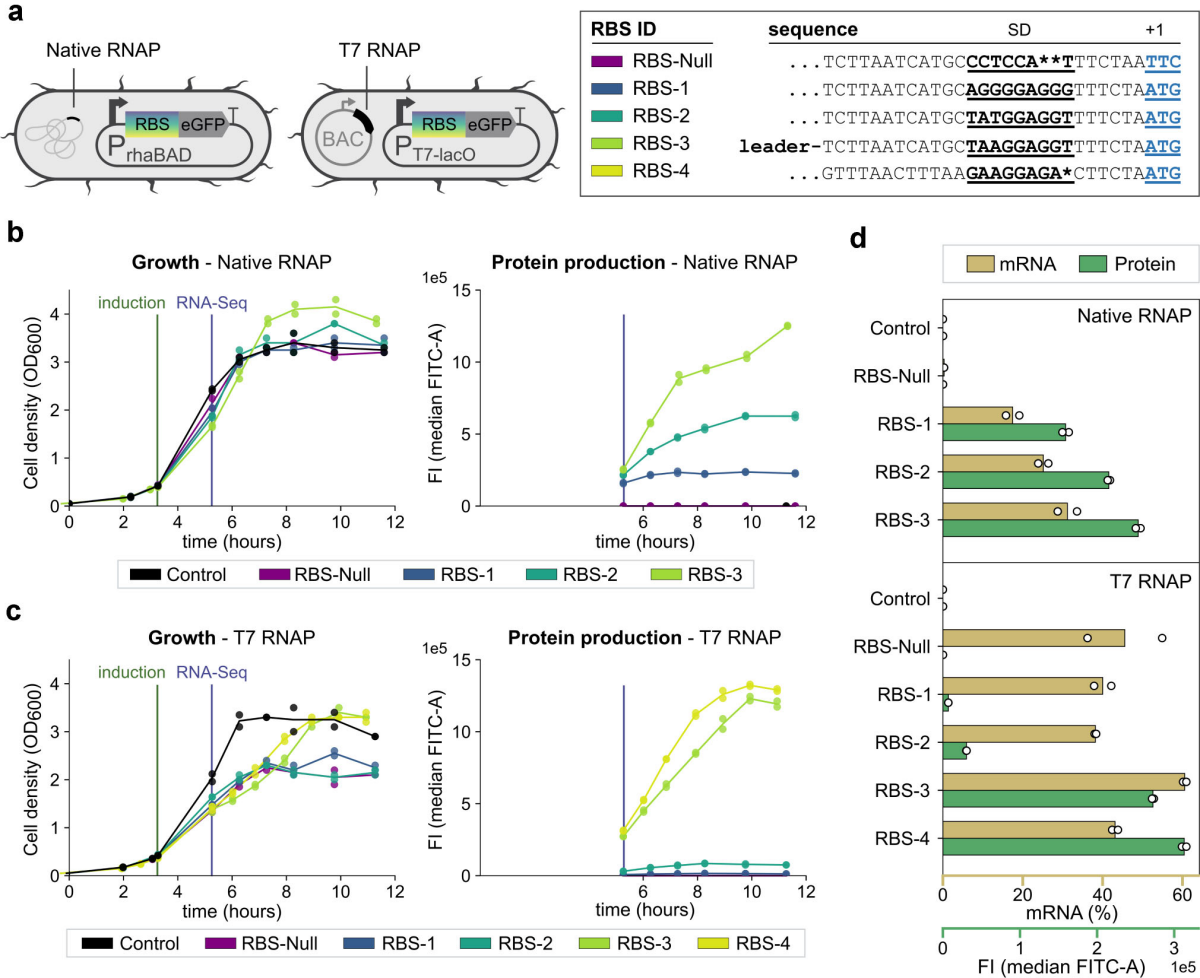
Decoupling transcription and translation using RBS variants. (**a**) Expression plasmid designs. eGFP was expressed using either the native RNAP or T7 RNAP (T7 gene from a bacterial artificial chromosome). Five RBS variants were used to modulate translation efficiency, shown here with their corresponding sequences. Growth (OD_600_, left) and protein production (fluorescence intensity; median FITC-A, right) over time for (**b**) native RNAP and (**c**) T7 RNAP systems. Cultures were induced at OD ∼0.40 (induction line) and RNA-seq samples collected 2 h post-induction (sampling line). (**d**) eGFP mRNA levels (percent of total transcripts) and protein levels (median FITC-A) for each expression condition.

**Figure 3.**
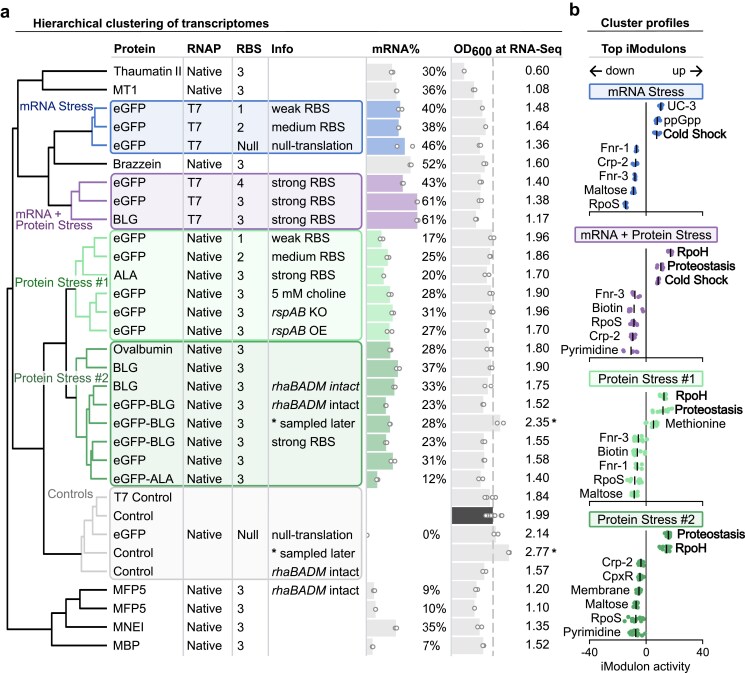
Hierarchical clustering reveals distinct transcriptional stress profiles. (**a**) Dendrogram of hierarchical clustering based on transcriptome similarity across all expression conditions. Each condition is annotated with the expressed protein, RNAP system, RBS variant, additional details (Info), heterologous mRNA% (percent of total transcripts), and cell density at RNA-seq sampling (OD_600_). The latter two are here visualized as horizontal bar charts overlaid with individual replicates. Samples were collected 2 h after induction at OD_600_ = 0.40. Samples marked with an asterisk were taken 3 h after induction. Five distinct clusters were identified: Control (gray), mRNA Stress (blue), mRNA + Protein Stress (purple), Protein Stress #1 (light green), and Protein Stress #2 (dark green). (**b**) iModulon activity signatures for each cluster. Strip plots show activities (scale: −40 to 40) of the top eight differentially activated iModulons per cluster; individual points represent single samples.

**Figure 4.**
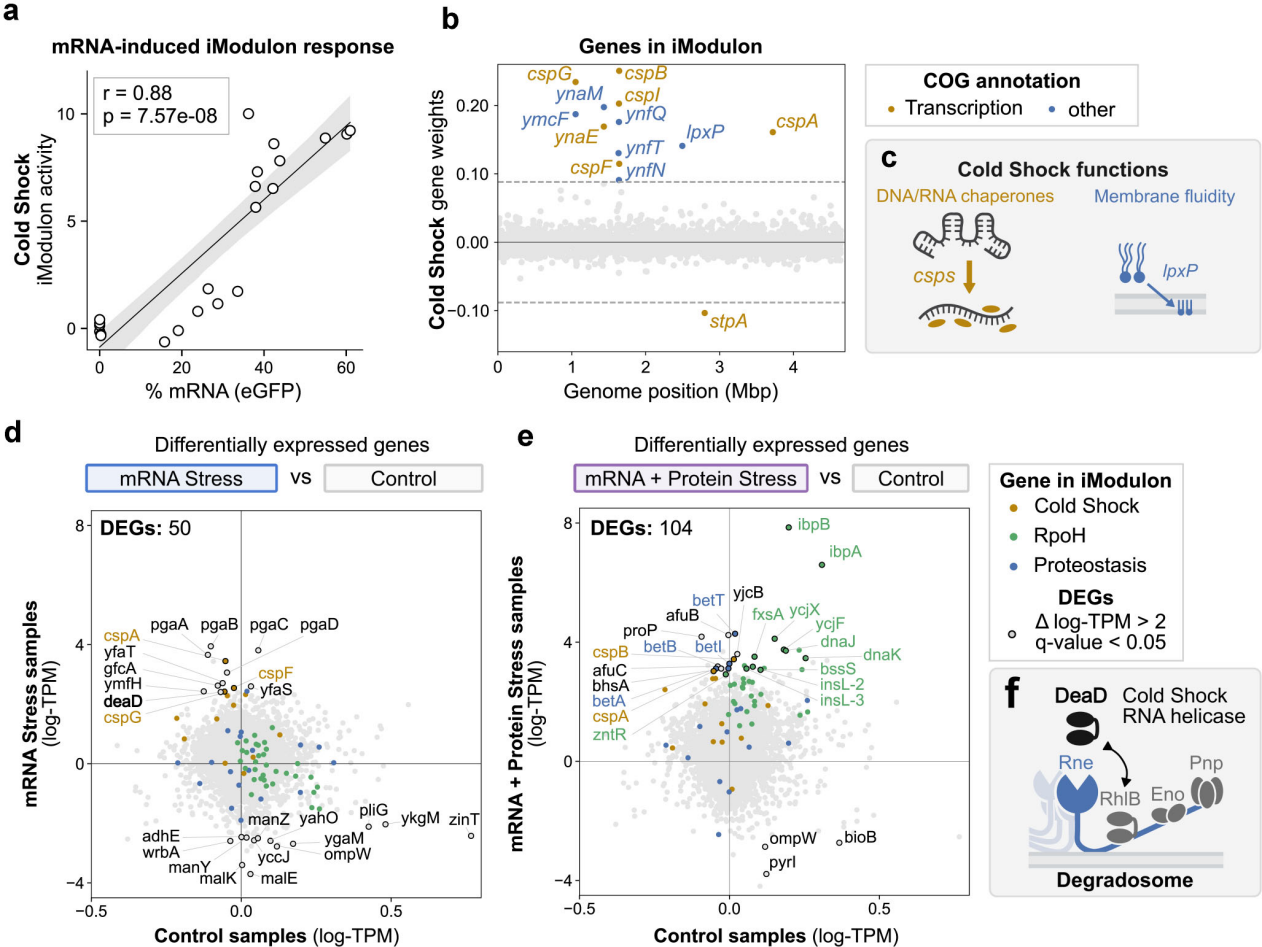
Cold shock as a signature stress response to transcriptional burden. (**a**) Correlation between *eGFP* levels (percent of total transcripts) across eGFP expression conditions. Pearson correlation coefficient (*r*) and *P*-value are shown; shaded region indicates 95% confidence interval. (**b**) Gene weights of Cold Shock iModulon genes plotted by position on genome. Positive weights indicate activation; negative weights indicate repression. Genes with weights above threshold are labeled and color-coded by COG functional annotation. (**c**) Schematic representations illustrating the cellular functions associated with cold shock response: DNA/RNA chaperones (CSPs) and membrane fluidity (LxpP). DEG scatterplots comparing samples in mRNA Stress cluster (**d**) and mRNA + Protein Stress cluster (**e**) to control cluster. Mean gene expression levels (log-TPM) in stress samples (*y*-axis) are plotted against control samples (*x*-axis). DEGs are defined by |Δ log-TPM| > 2 and *q*-value < 0.05. The top 25 most differentially expressed DEGs are outlined and labeled. Genes are colored by iModulon membership (Cold Shock: yellow, RpoH: green, Proteostasis: blue). (**f**) Schematic of the degradosome complex highlighting the cold shock RNA helicase DeaD.

**Figure 5.**
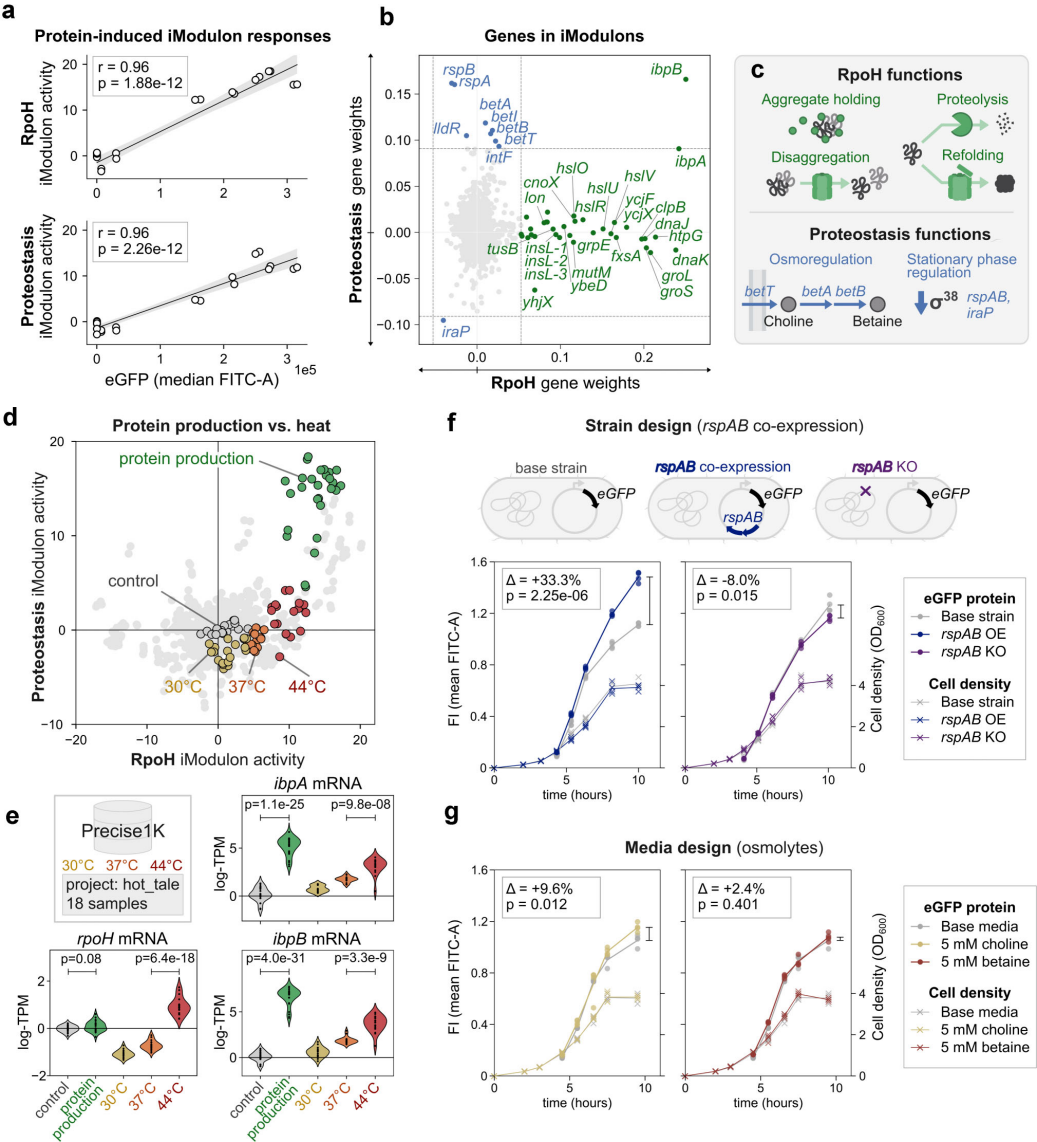
Characterizing protein production stress responses and engineering strain and media improvements. (**a**) Scatterplots showing the correlation between *eGFP* protein levels (median FITC-A) and iModulon activities for RpoH (top) and Proteostasis (bottom). Pearson correlation coefficients (*r*) and *P*-values are shown; shaded regions indicate 95% confidence intervals. (**b**) Gene membership comparison between RpoH and Proteostasis iModulons. Gene weights are plotted for each iModulon (*x*-axis: RpoH; *y*-axis: Proteostasis). Dashed lines indicate membership thresholds. Labeled genes exceed membership thresholds in their respective iModulons (green: RpoH genes; blue: Proteostasis genes). (**c**) Function of RpoH and Proteostasis iModulon genes. RpoH genes provide chaperone activity, disaggregation, proteolysis, and refolding. Proteostasis genes regulate osmotic stress and stationary phase adaptation. (**d**) Phase-plane plot comparing RpoH (*x*-axis) and Proteostasis (*y*-axis) iModulon activities across PRECISE-1K samples (*n* > 1000; see “Materials and methods” section). Samples from heat shock experiments (yellow: 30°C; orange: 37°C; red: 44°C) and Protein Stress clusters #1 and #2 (green) are highlighted; remaining samples are gray. (**e**) Violin plots comparing mRNA levels (log-TPM) of *rpoH, ibpA*, and *ibpB* across control, protein production, and heat shock conditions. Statistical comparisons are shown with *P*-values (Student’s *t*-test). (**f**) Effect of *rspAB* modulation on eGFP protein production. Top schematics show design of base strain, *rspAB* overexpression (OE), and *rspAB* knockout (KO). Time courses show development of eGFP production (mean FITC-A, circles, left *y*-axis) and cell density (OD_600_, crosses, right *y*-axis). (**g**) Effect of media supplementation with osmolytes (5 mM choline or 5 mM betaine) in eGFP-producing strains. Mean percent changes (Δ) and *P*-values (Student’s *t*-test) are used in panels (**f**) and (**g**) to compare eGFP levels from final timepoint samples to base controls.

**Figure 6.**
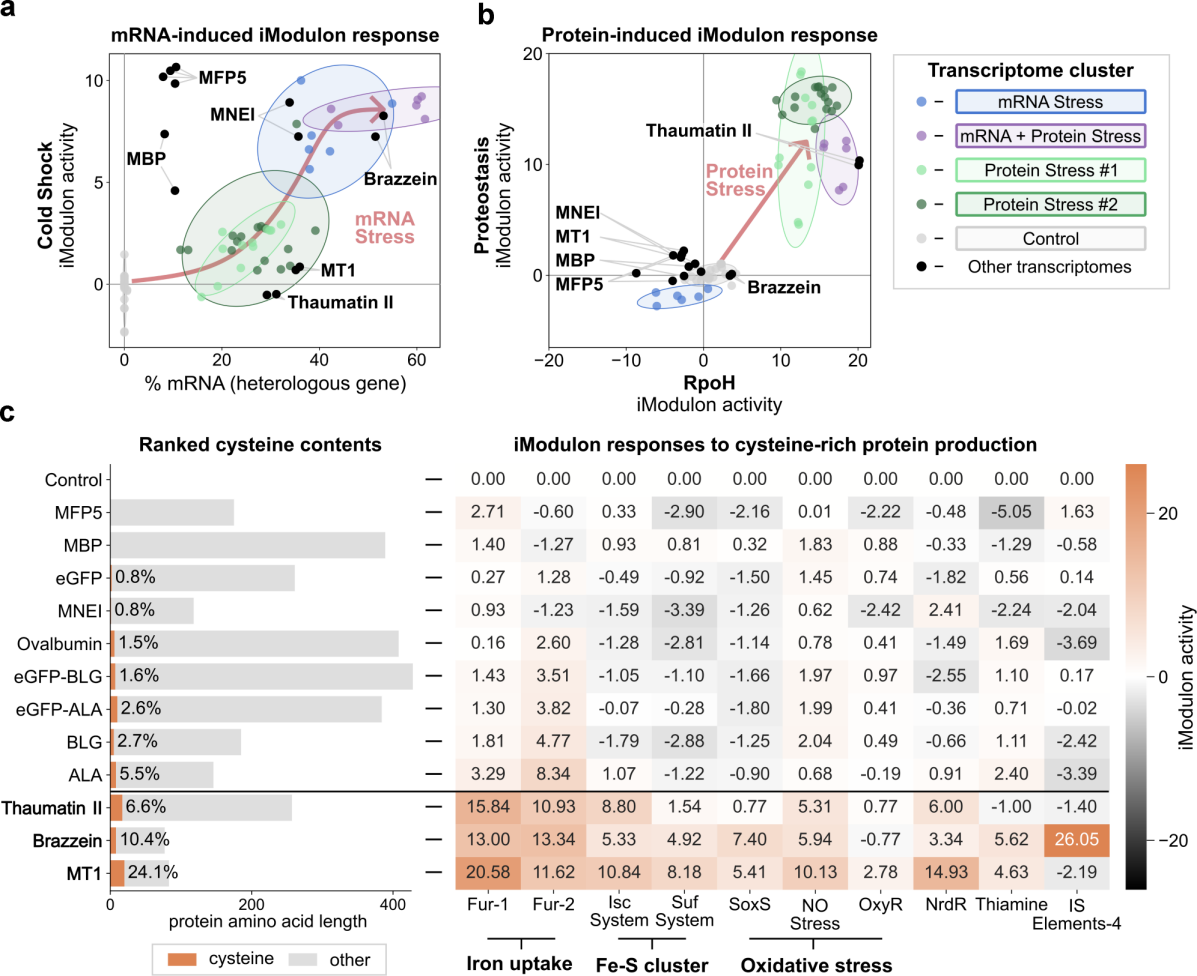
Product-specific cellular stress responses. (**a**) Cold Shock iModulon activity (*y*-axis) versus heterologous mRNA (*x*-axis, % of total transcripts). Colored ellipses represent the five main transcriptome clusters (identified in Fig. 3). An arrow indicates the trend of the Cold Shock response to increasing heterologous mRNA levels. Labeled points indicate individual expression conditions outside these clusters. (**b**) Proteostasis iModulon activity (*y*-axis) versus RpoH iModulon activity (*x*-axis). Colored ellipses represent the five main transcriptome clusters. An arrow indicates the direction of RpoH and Proteostasis iModulon activities to increasing protein stress. Labeled points show individual conditions. (**c**) Left: Horizontal bar chart with protein lengths and cysteine content (%) for all expressed proteins, ranked by cysteine percentage. Right: Heatmap of average iModulon activities across expression conditions with activities displayed numerically in each cell. These iModulons are related to iron regulation (Fur-1, Fur-2), Fe-S cluster assembly (Isc System, Suf System), oxidative stress (SoxS, NO Stress, OxyR), NrdR, thiamine biosynthesis, and transposon activity (IS Elements-4).

In the following sections, we first describe transcription decoupling from translation (Fig. 2), then present the distinct stress responses associated with transcriptional burden and protein production (Figs 3 –5). Finally, we examine product-dependent responses, including responses to cysteine-rich proteins (Fig. 6).

### Decoupling transcription from translation in native- and T7 RNAP expression systems

High transcription loads can impose a severe burden on *E. coli* production hosts, especially in T7 RNAP-based expression systems. To discriminate the burden caused by transcription or translation, we constructed nine different transcription/translation combinations of eGFP. Two transcription systems were used: a native RNAP-based rhamnose-inducible expression system (pNIC28-Bsa4 backbone) and a T7 RNAP-based expression system (pET-52B backbone). Translation levels were tuned by modifying RBS elements (Fig. 2a). Our resulting RBS library spanned from weak to strong variants, including a null-RBS variant with all ATG start codons replaced, and a control with the expression cassette removed entirely. RBS-3 featured a bicistronic design with one RBS driving a short leader peptide and a second RBS within this leader peptide driving eGFP [29].

We monitored cell growth (OD_600_), and eGFP production by fluorescence intensity (flow cytometry, median FITC-A). In the native RNAP system, eGFP production was easily modulated by tuning RBS strength (Fig. 2b). In contrast, the T7 RNAP system reduced growth and showed limited tunability of protein production by RBS strength (Fig. 2c). Over time, a growing subpopulation of T7 hosts lost fluorescence, likely due to instability of the bacterial artificial chromosome used for expressing the T7 RNAP gene (Supplementary Fig. S1).

We then investigated how eGFP protein levels related to *eGFP* mRNA levels (Fig. 2d). mRNA levels were defined as percentage relative to total mRNA (Methods). In the native RNAP system, levels of mRNA were strongly influenced by RBS strength. Conversely, the T7 RNAP system produced high mRNA levels regardless of RBS strength. The null-translation RBS variant showed very low mRNA levels (<1%) under native RNAP but maintained high levels (>38%) under T7 RNAP.

These results demonstrated how transcription can be decoupled from translation in a T7 RNAP expression system, but not when the gene is transcribed by the native *E. coli* RNAP. The native RNAP is physically coupled to ribosomes [35], and mRNA bound by ribosomes is protected from degradation by the degradosome [36]. Poorly translated mRNA is degraded rapidly [13, 37], as we demonstrated across our transcripts with weak RBS variants. In contrast, T7 RNAP transcription is approximately eight-fold faster than native transcription [19, 20], and can happen orthogonally to native transcription such that heterologous gene expression does not have to compete for binding to the native RNAP. Importantly, T7 transcription is not synchronized with translation [38], meaning transcripts can fold into secondary structures before engaging with the ribosome.

### Expression host transcriptomes cluster based on transcriptional burden and type of protein product

To identify stress responses to protein production, we obtained transcriptomes during active production of 12 different proteins. All proteins were expressed using the native RNAP system with a strong RBS (RBS-3), and production was confirmed via western blotting (Supplementary Fig. S2). RNA-seq samples were collected 2 h post-induction, with two conditions further sampled at 3 h. A complete view of growth profiles and timepoint sampling is provided in Supplementary Fig. S3.

To gain a broad view of how different expression conditions affected the host transcriptome, we analyzed global gene expression patterns. Hierarchical clustering (Ward’s method) of all unique expression samples revealed five distinct transcriptome clusters (Fig. 3a). Control samples, where no transgene was expressed, formed a separate cluster (“Controls”). T7 RNAP expression samples two distinct clusters based on RBS strength: one with high mRNA levels but poor translation (“mRNA Stress”), and another with high levels of both mRNA and protein (“mRNA + Protein Stress”).

The remaining clusters comprised samples expressing the 12 different heterologous proteins under the native RNAP system. Most proteins elicited similar transcriptomic responses, grouping them into two clusters (“Protein Stress #1″ and “Protein Stress #2″). These included the globular whey and egg white proteins α-lactalbumin (ALA), β-lactoglobulin (BLG), ovalbumin, the β-barrel protein eGFP, and fusion constructs (eGFP-ALA and eGFP-BLG). A subset of proteins elicited distinct transcriptomic profiles. These outliers included proteins that fold well (MBP, MNEI) as well as proteins with amino acid bias (thaumatin II, brazzein, MT1, and MFP5). Expression of MT1, MFP5, and most notably thaumatin II, was associated with reduced host growth (OD_600_, Fig. 3a). Altogether, these clustering patterns showed that growth and global transcriptomic patterns are influenced by transcriptional burden and the specific protein characteristics.

### Transcriptional burden and protein production activate different cellular stress responses

To characterize the cellular stress responses that define the observed transcriptomic changes, we identified the top differentially activated iModulons in each hierarchical cluster (Fig. 3a), focusing on those with elevated activity during heterologous gene expression.

Expression of most proteins (clusters: Protein Stress #1 and #2) was characterized by high activity of the RpoH iModulon (Fig. 3b). This response includes genes involved in the heat shock response that assist in refolding or degrading misfolded proteins. In parallel, another iModulon was also activated that we refer to as the Proteostasis iModulon. Genes in this iModulon serve broader adaptive functions and are detailed later.

In contrast, expression from the T7 RNAP system led to elevated activity of the Cold Shock iModulon (Fig. 3b). This response destabilizes mRNA secondary structures via cold shock proteins (CSPs) [39, 40]. A key observation was the difference between samples with poor translation efficiency (cluster: mRNA Stress) and those with protein production (cluster: mRNA + Protein Stress). While the Cold Shock iModulon was activated in both clusters, RpoH and Proteostasis iModulons were not activated in expression systems with poor translation. These results suggests that transcriptional burden contributes to a Cold Shock iModulon response, while protein production contributes to RpoH and Proteostasis iModulon responses.

### Cold shock is a signature cellular stress response to transcriptional burden

We compared *eGFP* mRNA levels from our transcription/translation library with Cold Shock iModulon activity and observed a significant correlation (*r* = 0.88, *P* = 7.57e10^−8^, Fig. 4a).

The Cold Shock iModulon mainly consists of CSPs *cspA, cspB, cspF, cspG, cspH*, and *cspI* [41], as well as adjacent uncharacterized genes (Fig. 4b). Other members include *lpxP*, a palmitoleoyl transferase that modifies lipid A to increase outer membrane fluidity in the cold [42], and downregulated *stpA*, a transcriptional regulator with RNA chaperone activity [43]. CSPs act as RNA and DNA chaperones that destabilize global mRNA secondary structures (Fig. 4c), and restore translation during acclimation to cold temperatures, where mRNA secondary structures form [39, 40].

To identify individually upregulated genes to transcriptional stress besides those in the Cold Shock iModulon, we performed DEG analysis. We compared transcriptomes from samples in the mRNA Stress cluster and the mRNA + Protein Stress cluster against those in the Control cluster (Fig. 4d and e). DEGs were identified using threshold settings of |Δ log-TPM| > 2 and *q*-value < 0.05 (Mann–Whitney U test, Methods). We identified 50 DEGs under the mRNA stress condition and 104 DEGs under mRNA and protein stress condition (Supplemental Data 5). The top 25 most DEGs in each comparison are labeled in Fig. 4d and e. mRNA stress activated cold shock genes, most notably *cspA*. The combined mRNA and protein stress further induced genes from the RpoH and Proteostasis iModulons, reflecting the added burden of protein production. Notably, both conditions showed a significant upregulation of an RNA helicase gene *deaD*. DeaD is known to play a role in stabilizing mRNAs that are inefficiently translated [44], including cspA mRNA [45]. DeaD substitutes RhlB and assembles into the degradosome, a membrane-associated protein complex that mediates mRNA decay during cold shock (Fig. 4f) [46].

Together, these findings point to a broader role of cold chock responses for managing transcriptional stress apart from cold stress. Previous reports showed *cspA* induction at 37°C by nutritional upshift [47], or growth phase transitions [45]. CSPs are regulated through rapid mRNA decay controlled by their RNA structure [48], rather than by a dedicated transcription factor, possibly making them sensitive to changes in transcriptional burden. The detailed mechanism linking high transcription to the cold shock response needs to be further investigated.

### Protein production triggers a heat shock response subset and a broad adaptation response

Protein production activated two parallel cellular responses: the RpoH and Proteostasis iModulons. We correlated the activities of these responses with eGFP fluorescence intensity levels from our expression library (Fig. 2d) and found significant positive correlations for both (*r* = 0.96 for both, RpoH: *P* = 1.88e-12, Proteostasis: *P* = 2.26e-12, Fig. 5a).

The heat shock response serves to remove denatured proteins to restore protein homeostasis and is controlled by the sigma factor RpoH (σ^H^) [49]. Although many genes are part of the RpoH regulon [50], their exact expression patterns depend on additional factors beyond RpoH itself [51]. Using ICA, we narrowed the broad RpoH regulon into a focused RpoH iModulon of 29 genes (out of ∼140 total). This subset included key heat shock proteins: proteases (*hslVU, hslO, lon, hspQ*), chaperones (*dnaKJ, groLS, grpE, htpG, cnoX*), the disaggregase *clpB*, and *ibpA* and *ibpB*, which sequester misfolded proteins [52] (Fig. 5b and c). Compared to the broader RpoH regulon, this iModulon is enriched for genes involved in chaperone activity and protein turnover (Supplementary Fig. S4). Notably, genes exclusive to the RpoH regulon (i.e. not included in the iModulon) showed low differential expression during protein production (Supplementary Fig. S4). This suggests that the RpoH iModulon captures the specific subset of heat shock genes that are transcriptionally responsive. Five additional genes were unique to the iModulon: three IS186 insertion element sequences (*insL-1, -2, -3*), the tRNA sulfur transfer subunit *tusB* [53], and the putative transporter *yhjX*.

In parallel, protein production stress also activated the Proteostasis iModulon, which consists of ten genes with diverse functions. Although this iModulon includes high gene weights for the heat shock proteins *ibpA* and *ibpB*, the remaining genes are unrelated to RpoH (Fig. 5b). Notably, it features the *betABIT* operon, which encodes genes involved in betaine synthesis, a potent osmolyte, from extracellular choline [54]. Since osmolarity is known to impact successful protein production [55, 56], the inclusion of this operon appears particularly relevant during protein production. Other members of the iModulon include the *rspAB* operon, which may repress the entry to stationary phase [57, 58] or be involved in glucuronate metabolism [59]. Additionally, *iraP*, which is downregulated in this response, encodes a protein used to stabilize RpoS during phosphate starvation [60]. We speculate that this may relate to the downregulation of RpoS observed in *E. coli* expression hosts [21].

To summarize, the heat shock response activated by protein production stress consists of a focused subset of genes from the RpoH regulon, enriched for chaperone and proteolysis functions. In addition, a parallel response is activated that balances osmolarity and may delay the entry into stationary phase.

### Transcriptional regulation differences between heat and protein production stresses

To uncover the regulatory basis of the RpoH and Proteostasis iModulons, we leveraged relevant transcriptome profiles from the PRECISE-1K RNA-seq database. This included a dedicated heat shock study (project: hot_tale [61]). We plotted RpoH versus Proteostasis iModulon activities across all (>1000) PRECISE-1K transcriptomes (Fig. 5d). As expected, RpoH activity increased with temperature (30°C, 37°C, and 44°C). However, unlike protein production, the Proteostasis iModulon was not strongly activated by heat treatment, highlighting it as a specific marker of protein production stress.

This prompted us to consider how RpoH itself is regulated. The stability of the RpoH sigma factor is mainly controlled by the availability of chaperones. Under non-stress conditions, chaperones inhibit and destabilize RpoH [62]. When misfolded proteins accumulate, these chaperones are sequestered away, increasing RpoH stability and thereby initiating the heat shock response. This response can therefore be activated through various ways that induce protein misfolding, such as ethanol, alkaline pH, hyperosmotic shock, and heterologous protein expression [17, 49].

However, a key regulatory distinction between heat and protein production stress lies in how they affect the transcriptional regulation of RpoH. *rpoH* mRNA acts as an RNA thermometer [62], so its level increases significantly in response to heat (Fig. 5e). In contrast, we did not find *rpoH* mRNA levels to significantly increase during protein production. Another regulatory mechanism involves IbpA, a small heat shock protein that represses translation of *rpoH* mRNA under non-stress conditions. This repression is alleviated when IbpA is recruited to sequester misfolded proteins [57]. We found that both *ibpA* and *ibpB* were significantly upregulated by protein production compared to heat treatment (Fig. 5c). In fact, *ibpA* and *ibpB* were by far the most upregulated genes during protein expression (Supplementary Fig. S4).

These results highlight the distinct mechanisms by which heat shock and protein production engage the heat shock response. Protein production does not significantly increase *rpoH* mRNA levels but induces very high *ibpAB* expression and uniquely activates the Proteostasis iModulon, which is not triggered by heat stress.

### Optimizing eGFP protein production guided by iModulon analysis

The beneficial role of the Proteostasis iModulon response on protein production was explored further through strain and media optimization. First, we examined the influence of the *rspAB* operon on eGFP production. In a positive control design, the *rspAB* operon was co-overexpressed downstream of the *eGFP* gene on the expression vector. In a negative control design, *rspAB* was knocked out in the production strain. Performances were evaluated at final timepoint samples. By overexpressing *rspAB*, production of eGFP increased significantly by 33% (Student’s *t*-test; *P* = 2.25e-06) with a slight reduction in growth (Fig. 5f). Meanwhile, the *rspAB* KO strain slightly reduced eGFP levels (−8%, *P* = .015). These results are consistent with previous findings showing that *rspAB* co-expression enhances β-galactosidase production in late-stage stationary fermentations [63].

To explore the underlying mechanism, we also investigated any DEG in the *rspAB*-overexpressing strain. Surprisingly, we observed no major changes in gene expression or RpoS iModulon activity (Supplementary Fig. S5). This suggests that the beneficial effect of *rspAB* may not involve global transcriptional regulation, as previously proposed [58].

Similarly, the *betABIT* operon also attracted our attention because betaine supplementation has been used to improve protein stability and yields [56] and alleviate a heat shock response [64]. To complement the observed upregulation of *betABIT* in our host, we tested the effects of supplementing either 5 mM choline or betaine at the induction time. Choline supplementation showed a significant increase in eGFP of 9.6% (*P* = .012), while betaine had no significant effect (Fig. 5g). Studies that successfully enhance protein production combine betaine supplementation with NaCl to stimulate betaine uptake [55, 56]. Betaine is taken up via transporters ProP or ProVWX, whose expression is induced by NaCl [65]. However, neither of these transporters was as highly upregulated as the *betABIT* operon during protein production (Supplementary Fig. S6). Based on the positive impact of choline (supported by *betT* expression) and neutral effect of betaine in medium, we hypothesize that supplementation of an osmolyte must be coordinated with the cellular regulation of transport systems to be taken up and benefit protein production.

### Cellular stress responses are influenced by the type of expressed proteins

The host transcriptome varies depending on the product being expressed (Fig. 3a). These changes are reflected in iModulon responses associated with transcriptional burden (Cold Shock iModulon) and protein production stress (RpoH and Proteostasis iModulons). Several products in our dataset activated the Cold Shock iModulon, including MFP5, MNEI, Brazzein, and MBP (Fig. 6a). Notably, this response was observed even in cases where mRNA levels were not excessive. For example, MFP5 strongly activated the Cold Shock iModulon despite accounting for only 10% of total mRNA. This suggests that specific features of the transgene may contribute to a cold shock response.

Not all heterologous proteins stimulated the RpoH or Proteostasis iModulons (Fig. 6b). This is likely because our tested proteins misfold to different extents. For example, MBP, native to *E. coli*, is well known for its stable folding and is commonly used as a solubility tag [33]. Similarly, the smaller proteins in our dataset, MT1 (kDa 8.6), brazzein (kDa 9.1), and MNEI (kDa 13.9), also did not activate the RpoH and Proteostasis iModulons. This suggests that the tendency of a target protein to misfold can be inferred from its transcriptional stress response through iModulons.

### Cysteine-rich protein expression activates responses regulating redox and iron homeostasis and oxidative stress

Proteins rich in cysteine residues are particularly difficult to express in the *E. coli* cytoplasm, since its reductive environment inhibits disulfide bond formation [66, 67]. Three of our expressed proteins had high levels of cysteine: Thaumatin II (6.6% cysteine), Brazzein (10.4%), and Metallothionein-1 (24.1%). Although these proteins did not form a distinct cluster in our global transcriptome analysis (Fig. 3a), their expression consistently activated iModulons linked to iron regulation, iron-sulfur cluster (Fe-S cluster) assembly, and oxidative stress responses (Fig. 6c).

Specifically, Fur-regulated iModulons (Fur-1, Fur-2) were upregulated during expression of cysteine-rich proteins, indicating disruption of iron homeostasis [68, 69, 70]. Even modest increases in cysteine content were reflected in elevated Fur-2 activity (Fig. 6c), highlighting the sensitivity of iron regulation. Cysteine-rich proteins also activated Fe-S cluster assembly iModulons, including both the housekeeping Isc system and the stress-responsive Suf system. Fe-S clusters serve as key redox sensors in maintaining redox balance and are involved in electron transfer and sulfur metabolism [71].

The expression of cysteine-rich proteins further triggered oxidative stress responses, including activation of the nitric oxide (NO Stress) and superoxide (SoxS) iModulons, while the OxyR iModulon remained inactive. The oxidative stress responses are linked to Fur regulation and the SoxS regulator is directly induced by Fe-S cluster oxidation [71]. Oxidative stress can compromise genomic integrity, and we also observed upregulation of the Fur-regulated NrdR iModulon, which controls DNA precursor synthesis and responds to oxidative stress [72, 73]. Brazzein further activated the complex IS Elements-4 iModulon, which contains numerous transposase genes (Supplementary Fig. S7). Since spontaneous transposition can be induced by oxidative stress [74] and is a known challenge in cysteine production hosts [75], we speculate that cysteine-rich protein expression may influence the occurrence of such events.

In summary, the expression of proteins with high cysteine content triggered a coordinated set of cellular stress responses involved in iron and redox regulation and oxidative stress. We speculate that this response either results from the depletion of cysteines, or from the toxic accumulation of cysteine residues that may disrupt the finely tuned redox regulatory network maintained by Fe–S clusters.

## Discussion

Predictable expression of heterologous proteins remains a challenge. While heterologous gene expression is known to impose a metabolic burden, the associated cellular stresses are not well understood. This study dissected and revealed distinct stress responses in *E. coli* associated with the transcription and translation of heterologous genes.

High transcriptional burden triggered a cold shock response that destabilizes mRNA secondary structures via CSPs and via the DeaD RNA helicase. Their ability to modulate mRNA structure has made these useful tools in protein production: CSPs can enhance yields in cell-free systems at low temperatures [76], and overexpression of *deaD* can increase yield per transcript in T7 RNAP systems [44]. Targeting the cold shock response may offer a promising route to enhance expression.

When expressed, most of our proteins activated a heat shock response that helps to refold and degrade misfolded proteins. Protein production induced a subset of heat shock genes, notably *ibpAB*, while *rpoH* mRNA levels remained unchanged. The heat shock response has been leveraged in many applications for protein production, including chaperone co-expression plasmids to increase product yields [77], stress-responsive feedback expression systems [78], and biosensors to monitor cellular stress [79].

In addition to the heat shock response, we also identified another distinct set of co-activated genes that were specifically induced by protein production. These included genes involved in osmoregulation (*betABIT*) and stationary phase regulation (*rspAB*). Future transcriptomic studies that activate these genes independently may clarify whether they belong to separate regulatory modules. Targeting their functions through strain and media modifications improved eGFP yields, suggesting a role in maintaining proteostasis during protein production. This aligns with previous studies that have enhanced protein production by adjusting osmolarity [55, 56] or by co-expressing *rspAB* [63]. The exact functional role of *rspAB* warrants further investigation, as we did not observe transcriptional changes from knocking out or overexpressing *rspAB* in our expression system.

Proteins that did not activate a typical stress response included small, stable products and those with extreme amino acid compositions. Notably, cysteine-rich proteins distinctly activate oxidative stress responses and functions to balance iron- and redox homeostasis. Strategies that modulate the redox environment may be promising to improve production of cysteine-rich proteins; it was previously shown that production of a cysteine-tagged protein could be improved by deletion of *oxyRS* [80]. Further studies on diverse protein types are needed to achieve a full understanding of the protein type:host response relationship.

This study demonstrated the strength of big data transcriptomic data analytics for revealing transcriptional, translational, and product-specific cellular stress responses in expression systems. These insights provide a systems-level basis for developing new strategies to improve protein production, and the approach may be extended to other host organisms.

## Supplementary Material

gkag256_Supplemental_Files

## Data Availability

Expression plasmids used in this study have been deposited to Figshare (DOI: 10.6084/m9.figshare.30920657, see Supplementary Data File 1 for plasmid metadata). Plasmids are available on Addgene under plasmid IDs 245803–245829. Raw RNA sequencing data files (FASTQ) and mapped gene expression data (log-TPM) are available at GEO accession GSE305476 (BioProject: PRJNA1306061). RNA-seq data processing and ICA were carried out using existing source code from the iModulonMiner workflow (https://github.com/sbrg/iModulonMiner, commit 825963d). The ICA object, containing gene expression data and associated iModulon activities for the combined RNA-seq datasets, is available on Figshare (DOI: 10.6084/m9.figshare.30126562.v1).

## Notes

Present address: 21st.BIO A/S, Søborg 2860, Denmark

Previous address: Novo Nordisk Foundation Center for Biosustainability, Technical University of Denmark, Kgs. Lyngby 2800, Denmark

Present address: Novo Nordisk Foundation Biotechnology Research Institute for the Green Transition, Technical University of Denmark, Kgs. Lyngby 2800, Denmark

Previous address: Novo Nordisk Foundation Center for Biosustainability, Technical University of Denmark, Kgs. Lyngby 2800, Denmark

Present address: Department of Microbiology and Immunology, The University of Melbourne, Parkville, Victoria 3010, Australia

Previous address: Novo Nordisk Foundation Center for Biosustainability, Technical University of Denmark, Kgs. Lyngby 2800, Denmark

Present address: Microbial Expression Engineering, Novo Nordisk A/S, Bagsværd 2880, Denmark

Previous address: Novo Nordisk Foundation Center for Biosustainability, Technical University of Denmark, Kgs. Lyngby 2800, Denmark

Present address: Novo Nordisk Foundation Biotechnology Research Institute for the Green Transition, Technical University of Denmark, Kgs. Lyngby 2800, Denmark

Previous address: Novo Nordisk Foundation Center for Biosustainability, Technical University of Denmark, Kgs. Lyngby 2800, Denmark

Present address: Novo Nordisk Foundation Biotechnology Research Institute for the Green Transition, Technical University of Denmark, Kgs. Lyngby 2800, Denmark

Previous address: Novo Nordisk Foundation Center for Biosustainability, Technical University of Denmark, Kgs. Lyngby 2800, Denmark

Previous address: Novo Nordisk Foundation Center for Biosustainability, Technical University of Denmark, Kgs. Lyngby 2800, Denmark

Present address: Novo Nordisk Foundation Biotechnology Research Institute for the Green Transition, Technical University of Denmark, Kgs. Lyngby 2800, Denmark

Previous address: Novo Nordisk Foundation Center for Biosustainability, Technical University of Denmark, Kgs. Lyngby 2800, Denmark
