# Supplementary material for: Dissecting host stress responses for predictable heterologous gene expression in E. coli
Source: Nucleic Acids Res. 2026 Mar 30;54(6):gkag256. doi: 10.1093/nar/gkag256 (PMC13034041; doi:10.1093/nar/gkag256)
Supplement: gkag256_Supplemental_Files [file gkag256_supplemental_files.zip › Supplementary Figures.pdf]

SUPPLEMENTARY FIGURES

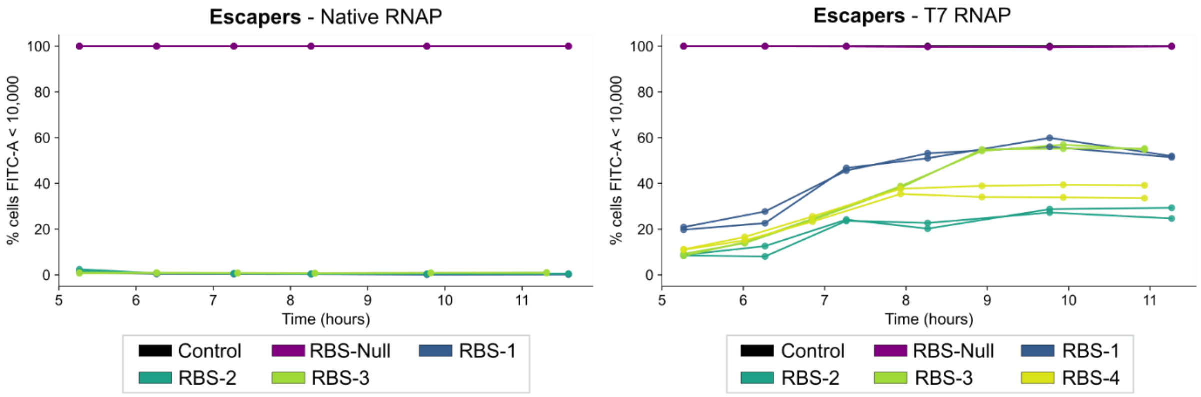

Supplementary Figure 1: Escaper cell populations in native RNAP and T7 RNAP expression systems. Fraction of non-fluorescent cells (Percent cells with FITC-A < 10,000) over time for native RNAP (left) and T7 RNAP (right) expression systems with variable RBS elements. In the native RNAP system, Control and RBS-Null shows no fluorescence (100% escapers) while other variants maintain stable fluorescent populations. In the T7 RNAP system, an increasing fraction of cells lost fluorescence over time across the RBS variants.

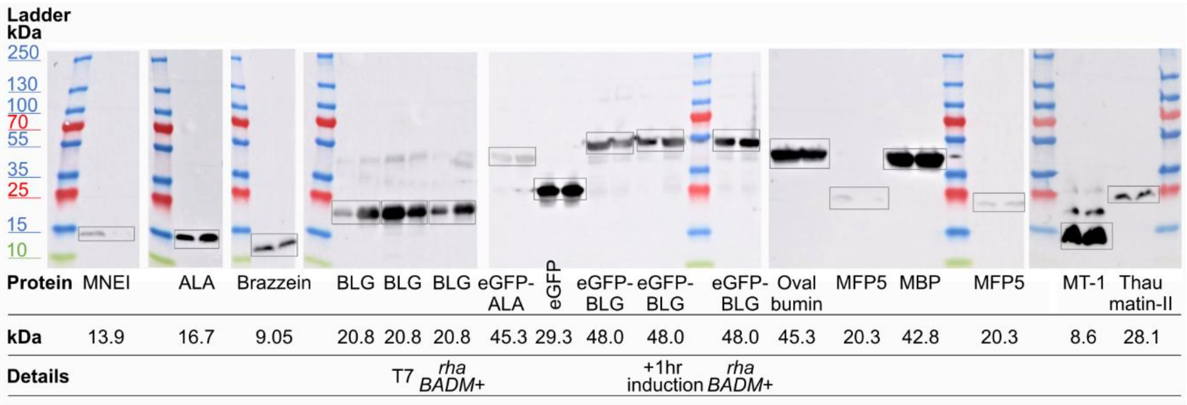

Supplementary Figure 2: Western blot of expressed proteins. Protein gels show protein ladder (left, kDa) and detection of heterologous protein expressions. Protein names and expected molecular weights (kDa) are indicated below each lane. “Details” specifies non-standard expression conditions: T7 indicates expression from the T7 RNAP-based system (pET-52b vector); all other proteins were expressed from the rhamnose-inducible system (pNIC28-Bsa4 backbone). *rhaBADM+* indicates expression in a strain with rhamnose metabolism genes intact (SDT492), other proteins were expressed in a strain with these genes knocked out (SDT507).

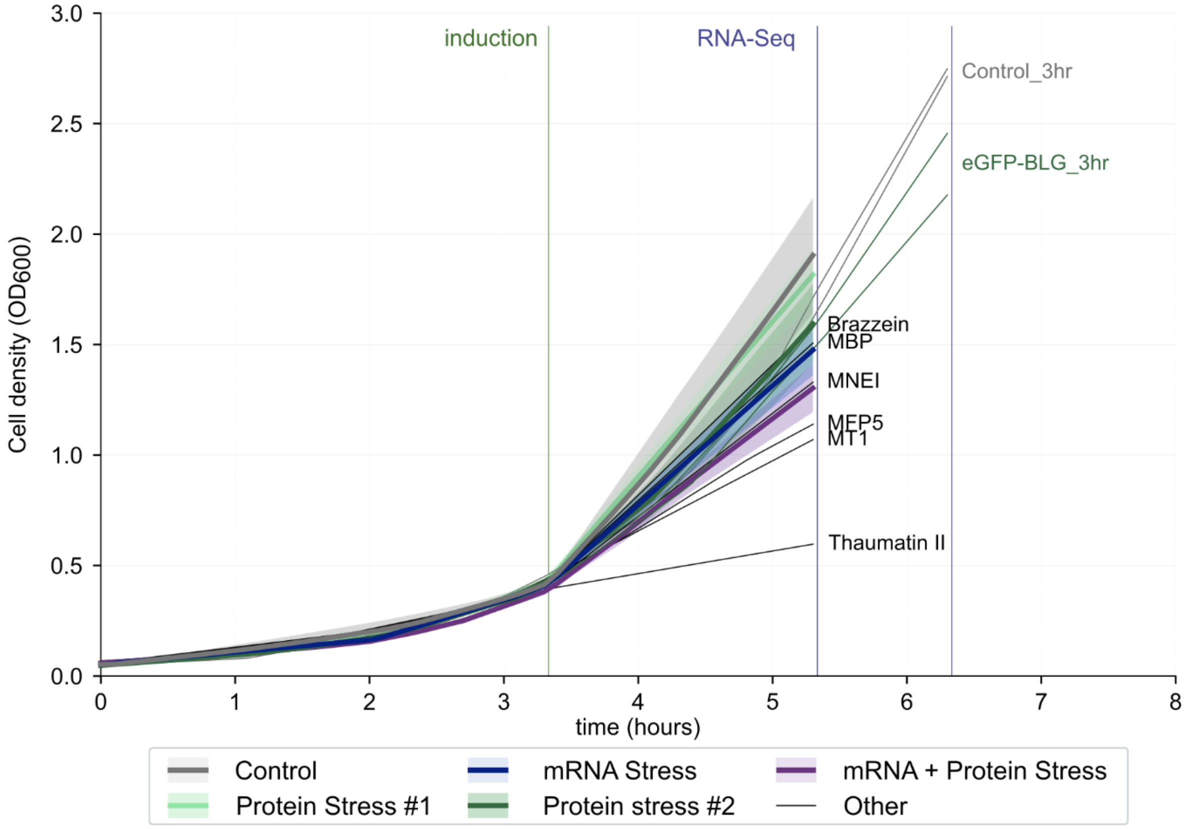

Supplementary Figure 3: Growth (cell density, OD_{600}) over time and induction and RNA-Seq sampling timepoints. The shown growth curves are time-shifted to align at induction time 3.23 hours (vertical green line). Colored lines and shaded areas represent cluster means ± standard deviation for the five transcriptome clusters (from **Figure 3**): Control (gray), mRNA Stress (blue), mRNA + Protein Stress (purple), Protein Stress #1 (light green), and Protein Stress #2 (dark green). Individual growth curves for outlier conditions (Thaumatin II, MT1, Brazzein, MFP5, MNEI, MBP) are shown as thin black lines. RNA-Seq samples were taken 2 hours post-induction, and extended samples were taken at 3 hours post-induction for Control_3hr and eGFP-BLG_3hr (vertical blue lines).

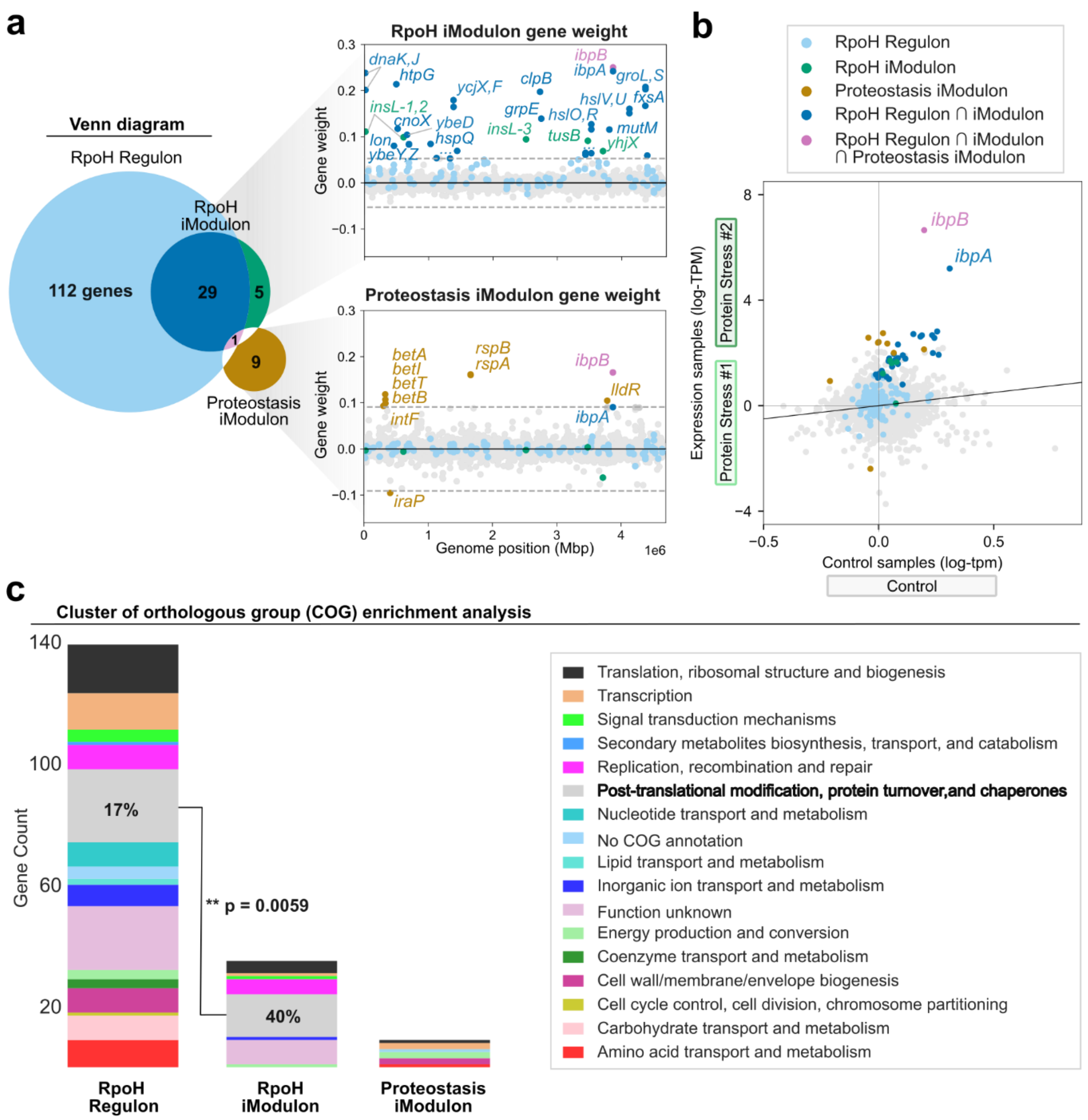

Supplementary Figure 4: Gene relationships between the RpoH regulon, RpoH iModulon, and Proteostasis iModulon. **a:** Venn diagram showing gene membership overlap between the RpoH regulon (142 genes), RpoH iModulon (29 genes), and Proteostasis iModulon (10 genes). Gene weight plots (right) show genes by genome position for RpoH iModulon (top) and Proteostasis iModulon (bottom). Genes above threshold are labeled and color-coded. **b:** Differential gene expression comparing protein production stress samples from Protein Stress #1 and #2 clusters (y-axis, log-TPM) versus control cluster samples (x-axis, log-TPM). Genes are colored by membership: RpoH Regulon only (light blue), RpoH iModulon (green), Proteostasis iModulon (yellow), genes in both RpoH Regulon and iModulon (dark blue), and genes in RpoH Regulon, iModulon, and Proteostasis iModulon (pink). **c:** Functional

enrichment analysis using Clusters of Orthologous Groups (COG) annotations. Stacked bars show the distribution of COG categories for RpoH Regulon (left), RpoH iModulon (middle), and Proteostasis iModulon (right). The RpoH iModulon is significantly enriched for "Post-translational modification, protein turnover, and chaperones" (40% of genes) compared to the broader RpoH Regulon (17%) ($p = 0.0059$, fisher's exact test).

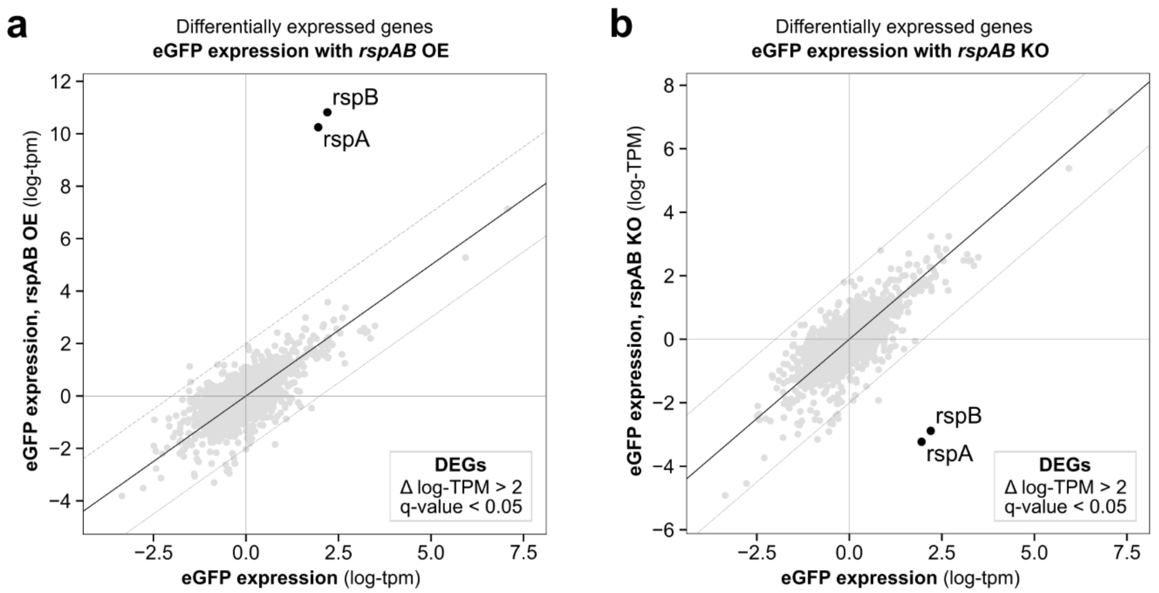

Supplementary Figure 5: Differential gene expression with overexpression (OE) or knock-out (KO) of the *rspAB* operon during eGFP expression. **a:** Differential gene expression comparing eGFP production (y-axis, log-TPM) with *rspAB* overexpression versus eGFP production alone (x-axis, log-TPM). **b:** Differential gene expression comparing eGFP production with *rspAB* knockout versus eGFP production alone. Gene expression in the *rspAB* KO strain (y-axis, log-TPM) is plotted against the base eGFP strain (x-axis, log-TPM). DEGs were defined by $|\Delta \text{ log-TPM}| > 2$ and q-value < 0.05. No DEGs were found.

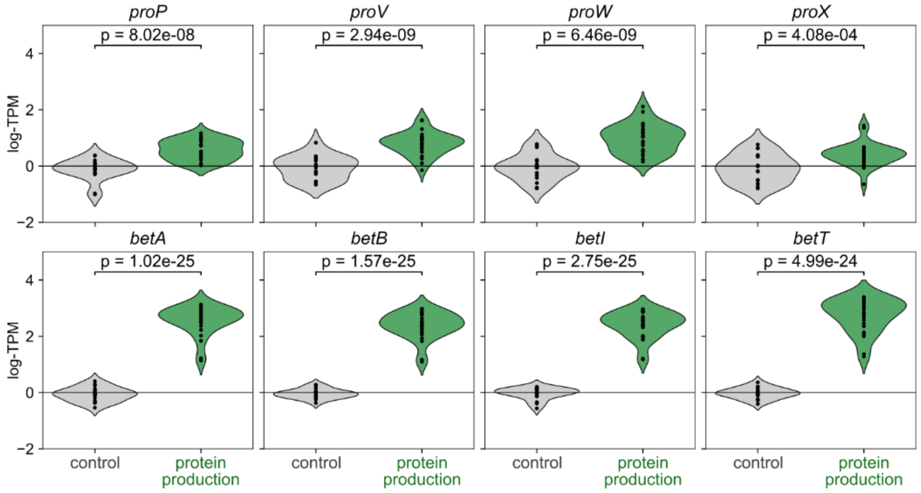

Gene	Δmean	p-value
proP	0.66	8.02e-08
proV	0.87	2.94e-09
proW	1.00	6.46e-09
proX	0.48	4.08e-04
betA	2.62	1.02e-25
betB	2.39	1.57e-25
betI	2.41	2.75e-25
betT	2.73	4.99e-24

Supplementary Figure 6: Differentially expressed betaine and choline transporter genes during protein production. Violin plots compare gene expression levels (log-TPM) between samples in control cluster and protein production cluster (Protein Cluster #1 and Protein Cluster #2). Top row: genes encoding transporters of betaine ProP (*proP*) and ProU (*proP*, *proV*, *proW*, *proX*). Bottom row: genes encoding choline transporter (*betT*) and other genes from the betaine biosynthesis operon (*betA*, *betB*, *betI*). Significant differential expression is indicated by p-values (student's t-test). Summary table (right) shows mean expression changes (Δmean) and p-values for each gene.

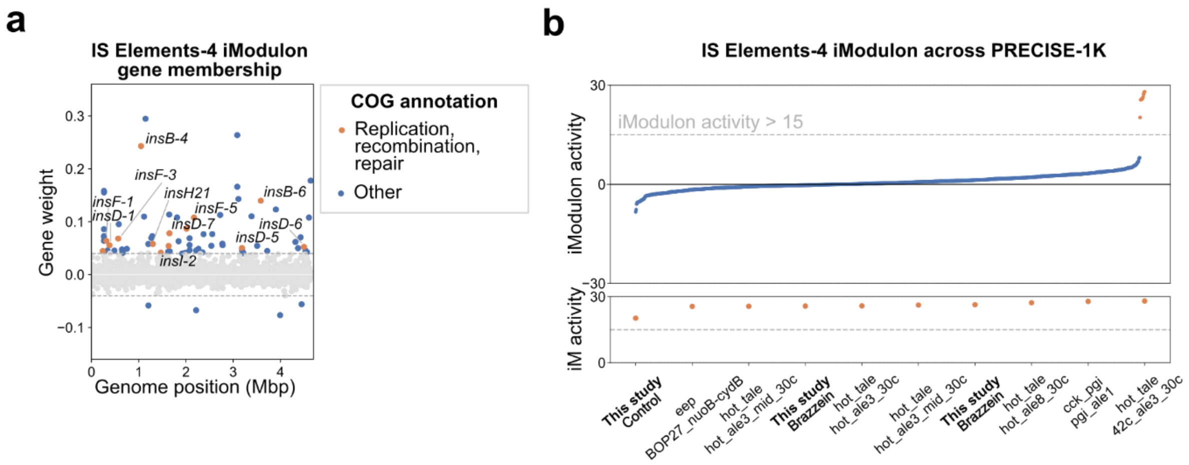

Supplementary Figure 7: The IS Elements-4 iModulon and its activity across different projects. **a:** Gene weights for IS Elements-4 iModulon plotted by genome position. Genes are colored by COG annotation: orange indicates "Replication, recombination, and repair" (11 genes, primarily transposases); blue indicates other functional categories (63 genes). **b:** IS Elements-4 iModulon activity across all PRECISE-1K samples (>1000 transcriptomes). Top: iModulon activity across all samples ordered by project; orange points indicate high activity (activity > 15). Bottom: iModulon activities for same samples with high iModulon activity.
